# Transforming growth factor beta 1 induced endothelin-1 release is peroxisome proliferator-activated receptor gamma dependent in A549 cells

**DOI:** 10.1186/s12950-016-0128-1

**Published:** 2016-06-10

**Authors:** Shulin Xiang, Yi Zeng, Bin Xiong, Yueqiu Qin, Xia Huang, Yujie Jiang, Weigui Luo, Suren R. Sooranna, Liao Pinhu

**Affiliations:** The First Clinical Medical College of Jinan University, Guangzhou, 510630 Guangdong Province China; Department of Intensive Care Unit, the People’s Hospital of Guangxi Zhuang Autonomous Region, Nanning, 530021 China; Department of Central Laboratory, Youjiang Medical University for Nationalities, Baise, 533000 Guangxi Zhuang Autonomous Region China; Department of Digestive Medicine, Youjiang Medical University for Nationalities, Baise, 533000 Guangxi Zhuang Autonomous Region China; Department of Respiratory Medicine, Youjiang Medical University for Nationalities, Baise, 533000 Guangxi Zhuang Autonomous Region China; Department of Surgery and Cancer, Imperial College London, Chelsea and Westminster Hospital, London, SW10 9NH UK; Department of Intensive Care Medicine, Youjiang Medical University for Nationalities, Baise, 533000 Guangxi Zhuang Autonomous Region China

**Keywords:** TGF-β1, PPAR-γ, Endothelin-1

## Abstract

**Background:**

Endothelin-1 (ET-1) is involved in pulmonary vascular remodeling. The aim of this study was to investigate the biochemical interactions between PPAR-γ, TGF-β1 and ET-1 in vitro.

**Methods:**

A549 cells were pre-treated with S2505 (10 μM), S2871 (10 μM) with/without SB203580 (10 μM) for 60 min following 2 h treatment with 10 ng/mL TGF-β1. A549 cells were also transfected with positive or negative PPAR-γ plasmids for comparison. RT-PCR, ELISA, western blotting and confocal laser scanning microscopy (CLSM) were used to measure the relevant expression of mRNA, protein, mediators of pathways and nuclear factor translocation.

**Results:**

SB203580 inhibited TGF-β1 induced ET-1 expression in A549 cells. S2871 decreased PPAR-γ mRNA and increase TGF-β1-induced ET-1 expression. S2871 increased phosphorylation of p38 MAPK and Smad2. Cells transfected with PPAR-γ negative plasmid increased TGF-β1 induced ET-1 expression, and increased the expression of phospho-p38 MAPK and phospho-Smad2. S2505 increased PPAR-γ mRNA expression, suppressed the increased TGF-β1-induced expression of ET-1. S2505 inhibited TGF-β1 induced phosphorylation of p38 MAPK and Smad2, also the nuclear translocation of Smad2. Cells transfected with PPAR-γ positive plasmid reduced TGF-β1-induced ET-1 expression, and inhibited the expression of phospho-p38 MAPK and phospho-Smad2.

**Conclusions:**

TGF-β1 induced release of endothelin-1 is PPAR-γ dependent in cultured A549 cells.

## Background

Pulmonary arterial hypertension (PAH) is a life-threatening illness characterized by increased pulmonary vascular resistance (PVR) following right heart dysfunction [1]. Several changes in the diagnosis and management of this disease have been implemented by the National Institute of Health (NIH) registry since the 1980s [2], but the outcome of this fatal disease although improved still remains poor [3, 4]. Recent research revealed that the median survival of PAH was between 3 and 5 years [5, 6].

The pathogenesis of PAH still remains elusive and there is general agreement that the endothelial dysfunction and pulmonary vascular remodeling appear to be the key prerequisite reasons for the initiation of the disease. Any stimuli leading to vascular endothelial injury, vasoconstriction, cell proliferation, proinflammatory, thrombogenic functions and vascular remodeling are likely to contribute to PAH [7, 8]. The increase in PVR is progressive and finally leads to right heart failure and death. Many factors may be involved in this progressive process and an understanding of molecular mechanisms of PAH has given rise to numerous lines of research and important discoveries in the last decade. The presence of inflammatory cytokines and increased expression of growth and transcriptional factors are thought to contribute directly to further recruitment of inflammatory cells and proliferation of smooth muscle and endothelial cells resulting in increased PVR [9]. ET-1, prostacyclin, TGF-β family and nitric oxide (NO) are closely related to pulmonary arterial smooth muscle cell (PASMC) proliferation [10].

ET-1 is a 21-AA peptide which regulates vasoconstriction and proliferative responses in numerous cell types and recent findings have re-established the role of ET-1 in the pulmonary vascular remodeling process. ET-1 plasma levels are prominently increased in PAH patients and correlate with PVR and PAH [10–13]. The concentration of ET-1 in pulmonary circulation correlated with the increased levels of PVR, as well as the severity of the structural abnormalities found in distal pulmonary arteries as measured by intravascular ultrasound [14]. Factors that can affect the expression of ET-1 will also affect pulmonary vascular remodeling. TGF-β1 is one of the multifunctional peptides that regulate proliferation, differentiation and other functions in several cell types. Increased expression of TGF-β1 has been observed in PAH vessel and contribute to PASMC growth and collagen deposition [15]. The effects of ET-1 leading to pulmonary vascular remodeling are enhanced by the presence of TGF-β1 in human PASMC [16]. The pathophysiology of pulmonary hypertension differs according to the presence or absence of lung disease. Idiopathic pulmonary fibrosis (IPF) is associated with a high incidence of pulmonary hypertension [17, 18]. Epithelial to mesenchymal transformation (EMT) of alveolar epithelial cells has been recognized as a potential contributor to IPF and TGF-β1 has a close relationship with EMT in A549 cells [19, 20]. Previous studies suggested that TGF-β1-induced A549 cells undergo EMT via phosphorylation of Smad2 [21, 22] and peroxisome proliferator-activated receptor gamma (PPAR-γ) ligands inhibited profibrotic changes in TGF-β1-stimulated cells [23, 24]. PPAR-γ is a ligand-activated nuclear receptor which regulates the transcription of genes involved in adipogenesis, insulin sensitization, inflammation, as well as vascular remodeling [25, 26]. Early research suggested that PPAR-γ activators inhibited oxidized low-density lipoprotein-induced induced ET-1 production in endothelial cells [27]. The expression of PPAR-γ was reduced in the pulmonary tissue of rat models of this disease [28] and pharmacological activation of PPAR-γ could effectively attenuate the upregulation of ET-1 signaling in mice or human pulmonary artery endothelial cells [29].

The way in which TGF-β1 and PPAR-γ regulate the expression of ET-1 and what signaling pathways participate in this process remain unclear. We hypothesize that TGF-β1 can stimulate A549 cells to produce ET-1 and that while PPAR-γ may has some effects on this progress. We measured the effects of TGF-β1 and PPAR-γ on ET-1 expression and production in A549 cells by using RT-PCR, ELISA, western blot and confocal laser scanning microscopy (CLSM).

## Methods

### Cell culture

Human type II alveolar epithelial cell line A549 was purchased from the American Type Culture Collection (VR-15™). The cells were propagated in Roswell Park Memorial Institute 1640 (RPMI 1640) media (Gibco, USA) supplemented with 10 % fetal bovine serum (FBS; Hyclone,USA), 1 % penicillin/streptomycin (Solarbio, China) and maintained at 37 °C in a humidified atmosphere of 95 % air: 5 % CO2. Cells were subcultured every 3–4 days when 70–80 % confluence was reached. For all experiments, A549 cells were seeded (5,000 cells/cm^2^) into six-well plates for total RNA isolation, into ninety-six-well plates for ELISA experiments, or into 60-mm dishes for protein extraction. Experiments were repeated at three independent times and performed in triplicate each time. Experiments were performed when a monolayer of A549 cells achieved 70–80 % confluence and cells were serum-deprived for 24 h before the drug treatments.

### Cell treatment and sample collection

The drug treatments consisted of 4 parts as follow: A. A549 cells were treated with TGF-β1 (Peprotech, USA) at 10 ng/mL, BMP-2 at 100 ng/mL, BMP-4 at 100 ng/mL and BMP-7 at 100 ng/mL respectively, bovine serum albumin (BSA) was used as a control. B. A549 cells were treated with SB203580 (Cell Signaling Technology, USA) and/or TGF-β1. The cells were pretreated with 10 μM SB203580 for 60 min before 10 ng/mL TGF-β1 stimulation while 0.1 % DMSO used was as vehicle control. C. A549 cells were treated with S2871 (Selleckchem, USA, USA) and/or TGF-β1. The cells were pretreated with 10 μM S2871 for 60 min before 10 ng/mL TGF-β1 stimulation while 0.1 % DMSO was used as vehicle control. D. A549 cells were treated with S2505 (Selleckchem, USA) and/or TGF-β1. The cells were pretreated with 10 μM S2505 for 60 min before 10 ng/mL TGF-β1 stimulation while 0.1 % DMSO was used as vehicle control.

The culture supernatants were harvested 12 h after drug treatment for ELISA measurements of ET-1. After incubated with agonists or antagonists for 2 h, total RNA was isolated from the cells by TRIzol Reagent (Invitrogen, USA) according to the manufacturer’s instructions. RNA integrity was checked electrophoretically and purity was quantified by using spectrophotometry. Cell lysate were collected with cell lysis buffer (P0013, Beyotime Biotechnology, China) with 1 μM phenylmethanesulfonyl fluoride (PMSF) at 0, 5, 10, 15 and 30 min after drug treatment respectively according to the experimental conditions for western blotting. Total protein concentration was measured via a spectrophotometer using the bicinchoninic acid (BCA) protein assay kit (P0010S, Beyotime Biotechnology, China) with BSA utilised as the protein standard. For immunofluorescence analysis by CLSM, the cells were fixed in 4 % paraformaldehyde for 30 min after TGF-β1 treatment for 15, 30 and 60 min respectively.

### Real-time PCR

After incubations with agonists or antagonists for 2 h, total RNA was isolated from the cells by TRIzol Reagent and reverse transcription was performed on 1 μg RNA with oligo (dT) primers in 20 μL reactions by using the PrimeScriptTM RT reagent kit with gDNA Eraser (Perfect Real Time) (TAKARA BIO INC, RR047A, Japan) according to the manufacturer’s instructions. Gene expression of ET-1, PPAR-γ and glyceraldehyde-3-phosphate dehydrogenase (GAPDH) was evaluated using real-time PCR and SYBR Green I dye (Bio-Rad, Hercules, CA). The primers of ET-1, PPAR-γ and GAPDH were obtained from Invitrogen (Life Technologies, Shanghai). The sequence of primers for the amplification of ET-1, PPAR-γ and GAPDH were as follows: ET-1 (human) forward: 5-CCAATCTTGGAACAGTCTTTTCCT-3, reverse: 5-GGACATCATTTGGGTCAACACTCC-3; PPAR-γ (human) forward: 5-ACTCCCTCATGGCAATTGAATGTC-3, reverse: 5-ATACTCTGTGATCTCCTGCACAGCC-3; GAPDH (human) forward: 5-GAAGGTGAAGGTCGGAGT-3, reverse: 5-GAAGATGGTGATGGGATTC-3. After 15 min of initial activation at 95 °C, PCR was carried out for 40 cycles at 94 °C for 15 s and 56.5 °C (ET-1) or 58.0 °C (PPAR-γ) for 30s. GAPDH was performed simultaneously and used as the housekeeping gene. The threshold cycle (Ct) value was measured, and the comparative gene expression was calculated by 2^−△△Ct^ method as described previously [30].

### ET-1 ELISA

Confluent cells were incubated with serum-free RM1640 for 24 h prior to drug treatment. TGF-β1, SB203580, S2871 and S2505 were added into the culture supernatant for 12 h and the supernatant was harvested and stored at − 80 °C. For the SB203580 + TGF-β1, S2871 + TGF-β1 and S2505 + TGF-β1 groups, SB203580, S2871 and S2505 were added into the supernatant respectively for 60 min then incubated with TGF-β1 for 12 h. All assays were performed in triplicates and the ET-1 protein concentration in the supernatants was measured using human endothelin-1QuantiGlo ELISA kit (R&D Systems, USA) according to the manufacturer’s instructions.

### Western Blotting

20 μg of total protein from each sample was separated by 10 % SDS polyacrylamide gels (SDS-PAGE). After electrophoresis, separated proteins were transferred onto the polyvinylidene fluoride (PVDF) membranes (Millipore, Billerica, MA, USA) and the membranes were blocked for 2 h at room temperature with 5 % BSA in TBST. Membranes were then probed with primary antibodies (1:10,000 for β-actin and 1:1000 for other antibodies) at 4 °C overnight. The antibodies used were phospho-p38 MAPK (Thr180/Tyr182) (D3F9) XP® Rabbit mAb (4511, Cell Signaling Technology), p38 MAPK (D13E1) XP® Rabbit mAb (8690, Cell Signaling Technology), phospho-Smad2 (Ser465/467) (138D4) rabbit mAb (3108, Cell Signaling Technology), Smad2 (D43B4) XP® Rabbit mAb (5339, Cell Signaling Technology), phospho-SAPK/JNK (Thr183/Tyr185) (81E11) rabbit mAb (4668, Cell Signaling Technology), phospho-NF-kB p65 (Ser536) (93H1) rabbit mAb (3033, Cell Signaling Technology), NF-kB p65 (C22B4) rabbit mAb (4764, Cell Signaling Technology) and beta-actin rabbit polyclonal antibody (#4967, Cell Signaling Technology). After washing with TBST, membranes were probed with secondary antibodies (anti-rabbit IgG, HRP-linked antibody, #7074, Cell Signaling Technology) for 2 h at room temperature. Immunoblots were visualised by enhanced chemiluminescence and the image of western blots was scanned by Quantity One software. Band densities were quantified with freeware image analysis software, NIH Image (National Institute of Health, Bethesda MD, USA). The results of phosphorylated protein were normalized against the intensity of the total protein in each sample. In some western blots beta-actin was used as a protein loading control.

### Plasmid transfection

A549 cells were allowed to grow till about 80 % confluency and then transfected with PPAR-γ positive or negative plasmid. The glycerol bacterial samples containing plasmids of human PPAR gamma sequence or small interfering RNA (siRNA) were synthesized by Invitrogen (Life Technologies, Shanghai). The glycerol bacterial samples were amplified in Luria-Bertani (LB) medium with 0.5 % amikacin at 37 °C overnight and the plasmid DNA extraction was performed using endofree maxi plasmid kit (TIANGEN BIOTECH, Beijing). The plasmid DNA was quantified by using spectrophotometry. Transfection was performed using LipofectamineTM 2000 reagent (Invitrogen) following the manufacturer’s instruction. Cells were incubated at 37 °C for 24 h in a humidified incubator containing 95 % air: 5 % CO_2_ and then treated with 10 ng/mL TGF-β1. Detailed methods are provided in the online supplementary data.

### Immunofluorescence analysis by confocal laser scanning microscopy (CLSM)

A549 cells were serum-deprived for 24 h before treatment after reaching 70–80 % confluence. TGF-β1 was added into the cultures at 10 ng/mL for 15, 30 and 60 min respectively. After treatment the culture supernatant was removed and the cells were washed with PBS 3 times and then fixed in 4 % paraformaldehyde for 30 min at room temperature, permeabilized by 0.1 % Triton X-100 in PBS for 20 min, blocked with 5 % BSA for 30 min. The cells were incubated with primary antibody of phospho-Smad2 rabbit mAb (3108, Cell Signaling Technology) at 4 °C overnight. After PBS washing for 3 times, secondary antibody (Alexa Fluor 488-labeled goat anti-rabbit IgG, Beyotime Inc.) was added to the cells and incubated in the dark at room temperature for 1 h. The cells were washed with PBS for 3 times and mounted with propidium iodide (PI)-containing mounting media (ZSGB-BIO, Beijing, China) for 5 min. After washing with PBS 3 times, the cells were observed and photographed by a confocal laser scanning microscope.

### Statistical analysis

Data are presented as means ± standard deviation of multiple determinations. Statistical analysis was performed using SPSS for windows (version 16.0, Chicago, USA). Differences between multiple groups were compared using One-way repeated measures ANOVA. The difference between a control and a treatment group or between two treatment groups was compared by two-sample Student’s *t*-test where stated in the figure legends. The difference was considered statistically significant at *P* < 0.05.

## Results

### TGF-β1 can promote A549 cells synthesis and secretion of ET-1

**Fig. 1:**
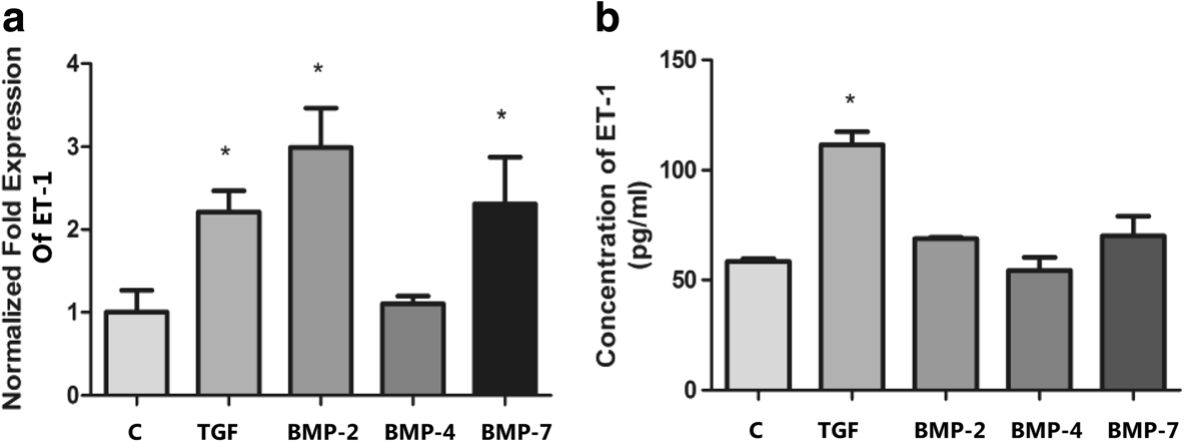
Effect of TGF-β1 on ET-1 in A549 cells. Confluent A549 cells were incubated with 10 ng/mL TGF-β1, 100 ng/mL BMP-2, 100 ng/mL BMP-4 and 100 ng/mL BMP-7 respectively. The incubation times were 2 h for real-time qPCR and 12 h for ELISA. The ET-1 mRNA expression is shown in Fig. 1a. The relative ET-1 mRNA levels (normalized to GAPDH mRNA) in TGF-β1, BMP-2 and BMP-7 group were increased than those in control group (*p < 0.05). The results of ELISA assay in Fig. 1b demonstrated that only the TGF-β1 treatment significantly increased the levels of ET-1 protein expression (*p < 0.05). The results are presented as means ± SD of 3 independent experiments performed in triplicate. (C, control group; TGF, TGF-β1 group; BMP-2, BMP-2 group; BMP-4, BMP-4 group; BMP-7, BMP-7 group)

Confluent A549 cells were incubated with 10 ng/mL TGF-β1, 100 ng/mL BMP-2, 100 ng/mL BMP-4 and 100 ng/mL BMP-7 respectively. After incubation for 2 h, the total RNA was extracted to perform real-time qPCR and after 12 h incubation the supernatants were harvested to evaluate ET-1 concentrations by ELISA. The results of real-time qPCR demonstrated that 10 ng/mL TGF-β1, 100 ng/mL BMP-2 and 100 ng/mL BMP-7 increased the relative ET-1 mRNA levels respectively in A549 cells but only the TGF-β1 treatment significantly increased the levels of ET-1 protein expression when measured by ELISA (Fig. 1).

### SB203580 suppressed the activation of MAPK P38 signal pathway and the expression of ET-1

**Fig. 2:**
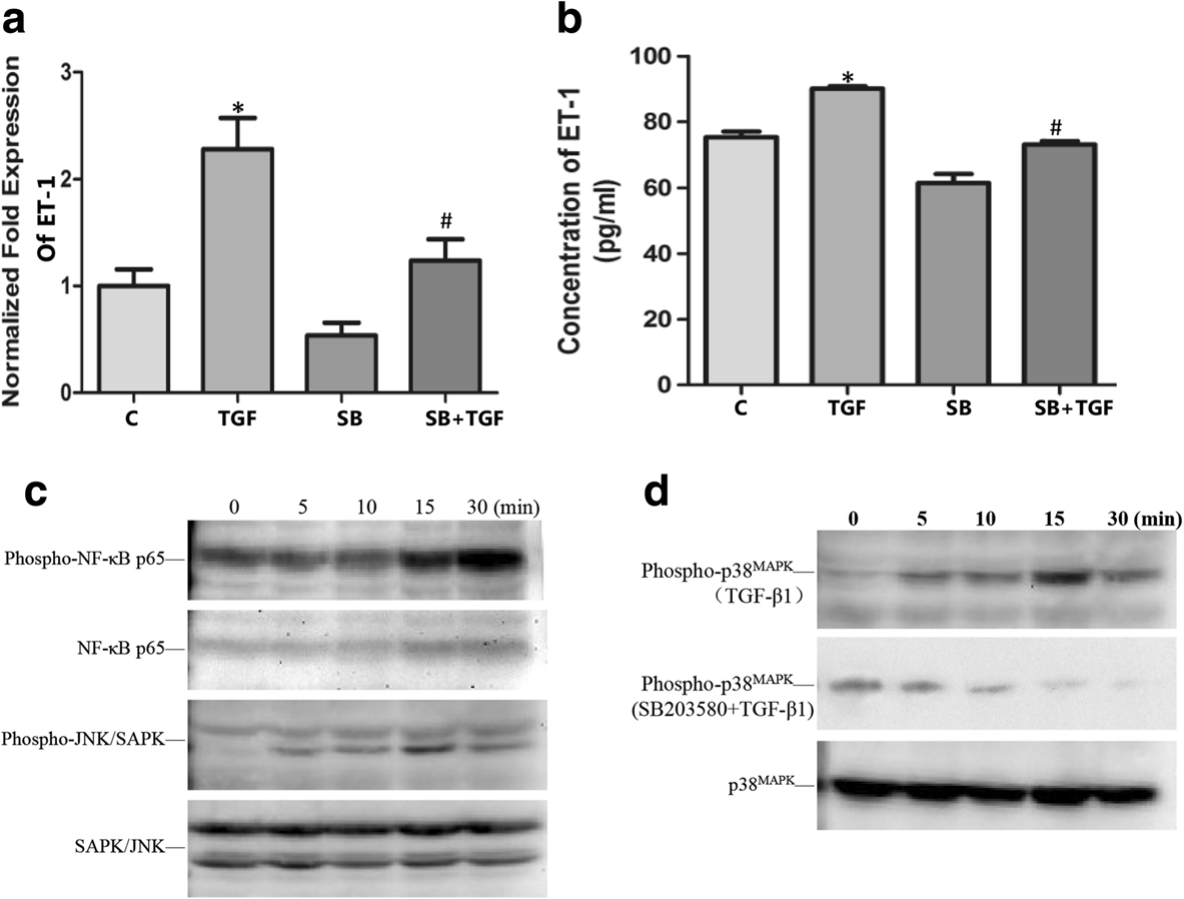
Effect of SB203580 on TGF-β1-induced up-regulation of ET-1 in A549 cells. Confluent A549 cells were treated with 10 ng/mL TGF-β1 or 10 μM SB203580 respectively while cells were pre-treated with 10 μM SB203580 for 60 min before 10 ng/mL TGF-β1 stimulation for SB203580 + TGF-β1 group. The samples were analyzed by real-time qPCR, ELISA and western blotting. The results of real-time qPCR were shown in Fig. 2a. The relative ET-1 mRNA level (normalized to GAPDH mRNA) in TGF-β1 group was significantly increased than those in control group (*p < 0.05). The relative ET-1 mRNA level in SB203580 + TGF-β1 group was decreased compared to TGF-β1 group (#*p* < 0.05). The results of ELISA assay in Fig. 2b also demonstrated the same results that TGF-β1 treatment increased the levels of ET-1 protein expression (*p < 0.05) and the combination of SB203580 could suppressed this effects (#*p* < 0.05). All the results are presented as means ± SD of 3 independent experiments performed in triplicate. Western blotting shows that TGF-β1 made the protein phosphorylation increased of NF-kB p65, JNK/SAPK (Fig. 2c) but the inhibitors of these pathways could not suppress the ET-1 protein expression according to the results of ELISA. TGF-β1 made the protein phosphorylation increased of p38 and SB203580 can effectively inhibit the expression of phospho-p38 (Fig. 2d). (C, control group; TGF, TGF-β1 group; SB, SB203580 group; SB + TGF, SB203580 + TGF-β1 group)

Confluent A549 cells were treated with 10 ng/mL TGF-β1 or 10 μM SB203580 (a p38 MAPK inhibitor) respectively while cells were pre-treated with 10 μM SB203580 for 60 min before 10 ng/mL TGF-β1 stimulation for the TGF-β1 + SB203580 group. The samples were then analyzed by real-time qPCR, ELISA and western blotting. The results of real-time qPCR and ELISA demonstrated that TGF-β1 increased the expression of ET-1 mRNA and protein. SB203580 can effectively block the TGF-β1 mediated ET-1 mRNA and protein increase. The results of western blotting demonstrated that TGF-β1 led to increased protein phosphorylation of NF-kB p65, JNK/SAPK and p38; SB203580 can effectively inhibit the expression of phospho-p38 mediated by TGF-β1 (Fig. 2).

### TGF-β1 mediated Smad2 phosphorylation and nuclear transfer

**Fig. 3:**
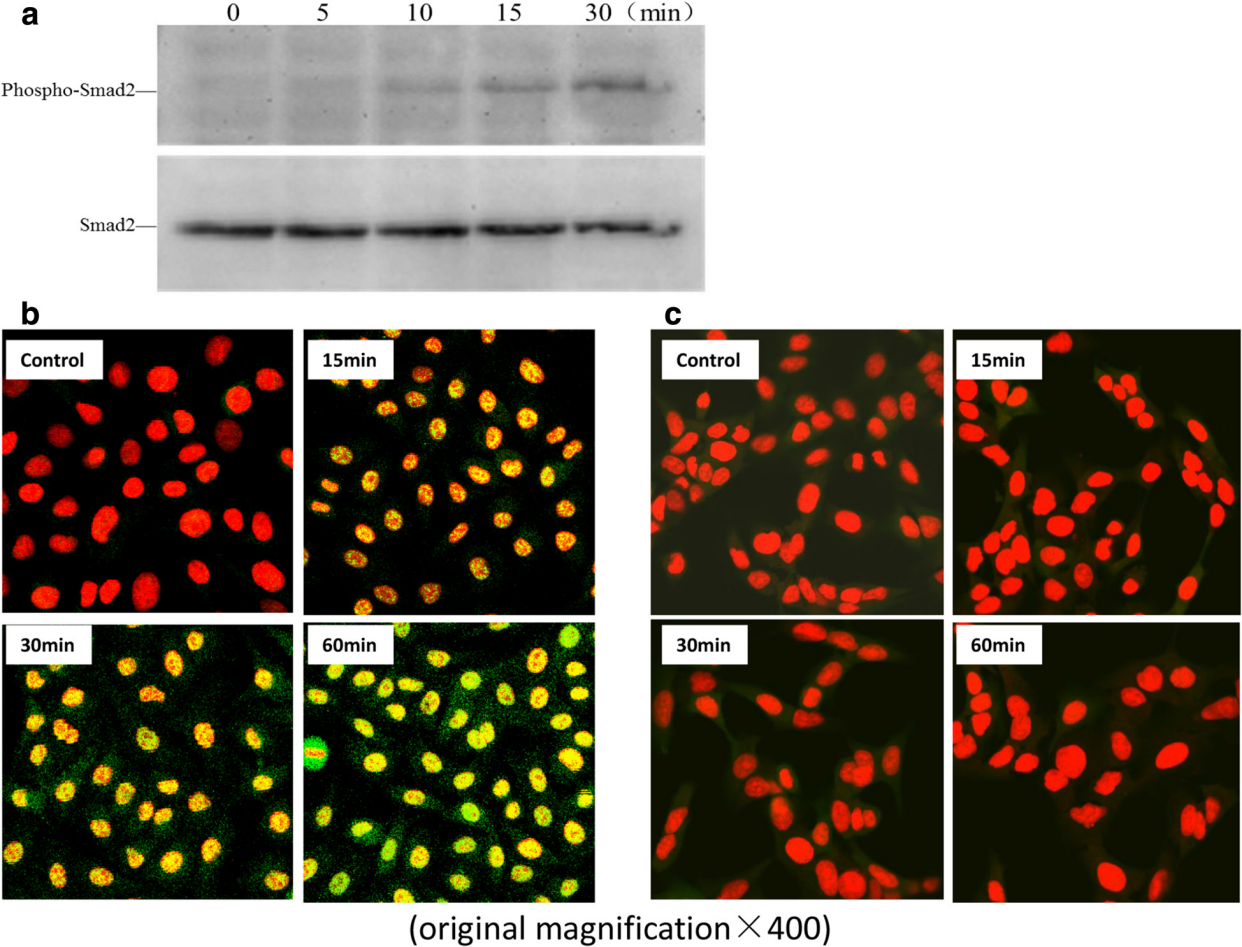
TGF-β1 mediated Smad2 phosphorylation and nuclear transfer. Confluent A549 cells were treated with 10 ng/mL TGF-β1 and cell lysates were subjected to western blotting analysis. For immunofluorescence analysis by CLSM, the cells were pre-treated without or with 10 μM SB203580 and then incubated with 10 ng/mL TGF-β1 and fixed in 4 % paraformaldehyde, treated as described in the Materials and Methods and the cells were observed and photographed by a confocal laser scanning microscope. The red fluorescence stained for cell nucleus and green fluorescence stained for phosphor-Smad2. Western blotting showed that TGF-β1 increased the phosphorylation expression of Smad2 (**a**). The pictures of CLSM demonstrated that TGF-β1 mediated Smad2 nuclear transfer (**b**), this process was suppressed by SB203580 (**c**) (original magnification × 400)

Confluent A549 cells were treated with 10 ng/mL TGF-β1 and total protein was extracts at 0, 5, 10, 15 and 30 min respectively for western blot analysis. For immunofluorescence analysis by CLSM, the cells were pre-treated without or with 10 μM SB203580 and then incubated with 10 ng/mL TGF-β1 for 15, 30 and 60 min respectively and nuclear transfer was observed by CLSM. The cell nuclei stained by PI were red in color and phosphor-Smad2 appeared green. The results of western blot suggested that TGF-β1 increased the phosphorylation expression of Smad2. The pictures of CLSM demonstrated that TGF-β1 mediated Smad2 nuclear transfer and this process was suppressed by SB203580 (Fig. 3).

### Inhibition of PPAR-γ can suppress the expression of PPAR-γ mRNA and increase the expression of ET-1 mRNA and protein mediated by TGF-β1, increase the expression of P-P38 and P-Smad2

**Fig. 4:**
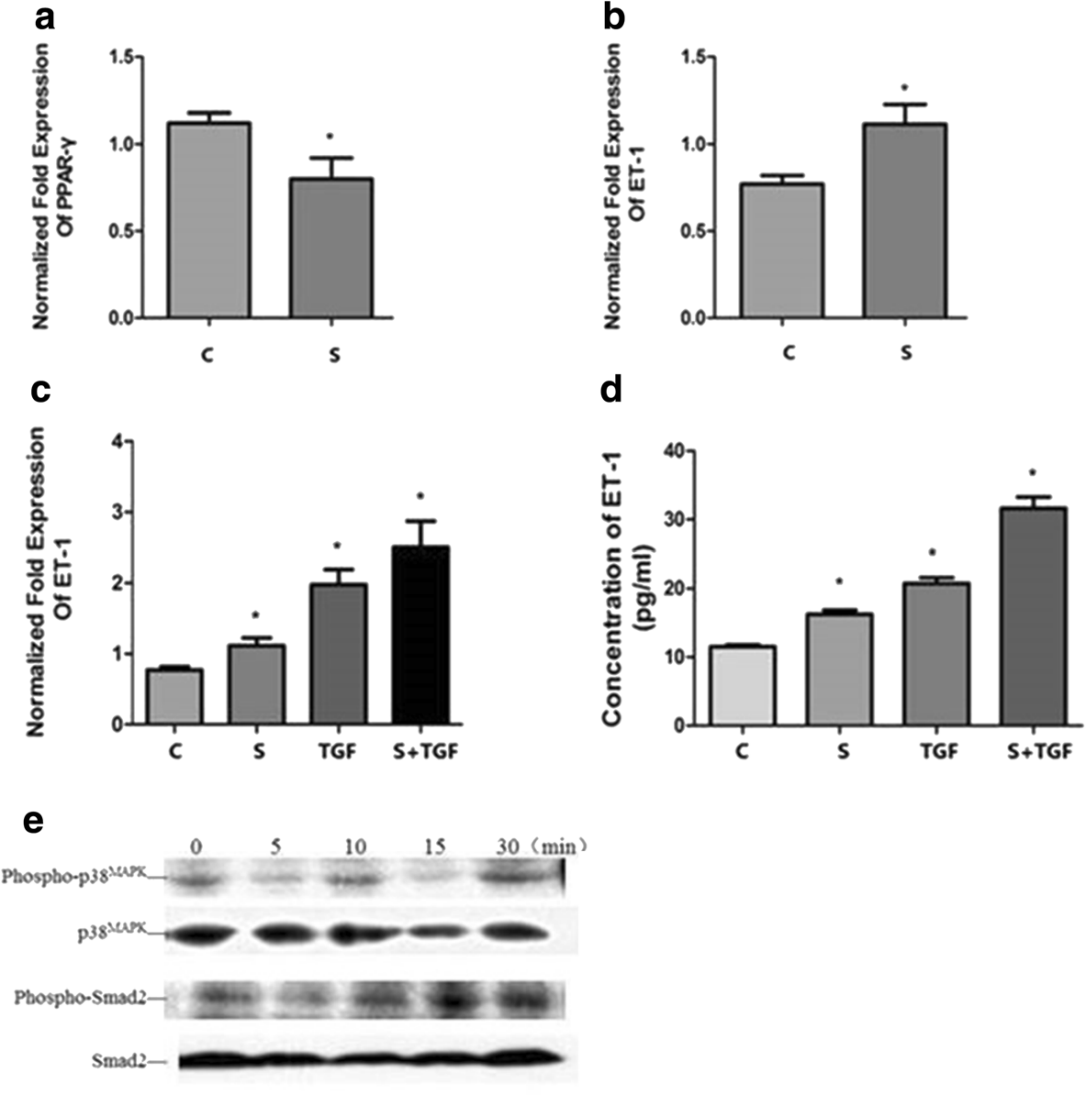
Inhibition of PPAR-γ on the expression of PPAR-γ and ET-1 mRNA. A549 cells were incubated with 10 ng/mL TGF-β1 or 10 μM S2871 respectively while cells were pre-treated with 10 μM S2871 for 60 min before 10 ng/mL TGF-β1 stimulation for S2871 + TGF-β1 group. Then the samples were collected and analyzed by real-time qPCR, ELISA and western blotting as described previously. The relative PPAR-γ mRNA level (normalized to GAPDH mRNA) in S2871 group was significantly decreased (**a**) while compared to control group and the ET-1 level was significantly increased (**b**) (*p < 0.05). The increased expression of ET-1 mRNA (**c**) and protein (**d**) mediated by TGF-β1was enhanced by S2871 (*p < 0.05). Western blotting showed that S2871 increased the expression of phospho-P38 and phospho-Smad2 (**e**). The results are presented as means ± SD of 3 independent experiments performed in triplicate. (C, control group; S, S2871 group; TGF, TGF-β1 group; S + TGF, S2871 + TGF-β1 group)

A549 cells were treated by the PPAR-γ inhibitor (10 μM S2871) with or without 10 ng/mL TGF-β1 and the samples were analyzed by real-time qPCR, ELISA and western blotting. The results demonstrated that S2871 suppressed the expression of PPAR-γ mRNA and increased the expression of ET-1 mRNA, enhanced the expression of ET-1 mRNA and protein mediated by TGF-β1 and increased the expression of phospho-P38 and phospho-Smad2 (Fig. 4).

### PPAR-γ silencing enhanced the expression of ET-1 mRNA and protein mediated by TGF-β1, increase the expression of phospho-P38 and phospho-Smad2

**Fig. 5:**
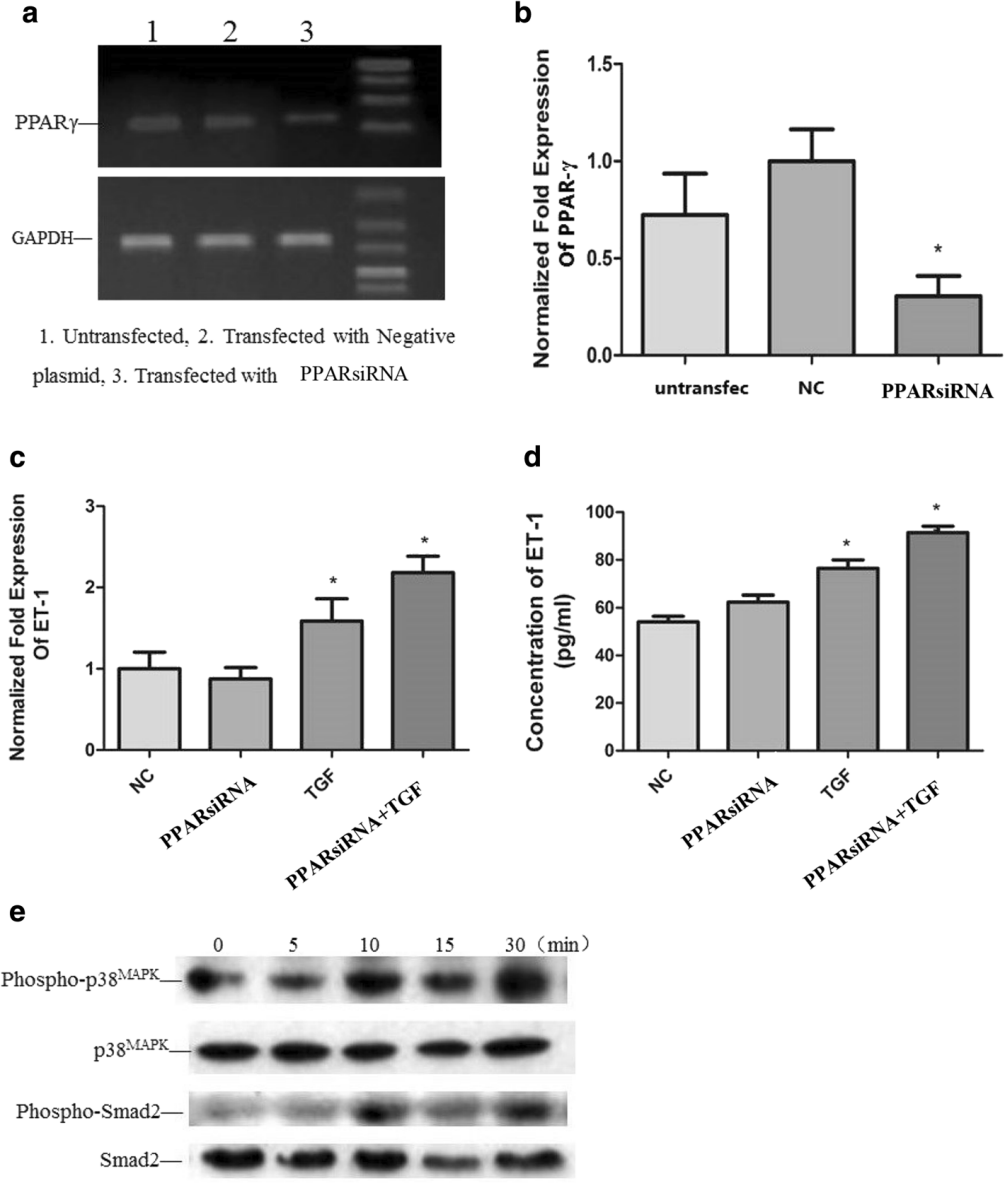
Silencing of PPAR-γ silence on the expression of ET-1. A549 cells were transfected with PPAR-γ siRNA plasmid or control vectors (*negative control*) using Lipofectamine^TM^ 2000 reagent and incubated at 37 °C for 24 h in an incubator containing 95 % air: 5 % CO2 and full humidity. Then transfected cells were treated with 10 ng/mL TGF-β1and the samples were collected and analyzed by real-time qPCR, ELISA and western blotting. The results of electrophoresis and real-time qPCR showed that PPAR-γ silencing reduced the expression of PPAR-γ mRNA (**a**, **b**) (*p < 0.05 while compared to NC). The results of RT qPCR (**c**) and ELISA (**d**) showed that PPAR-γ silencing slightly decreased the expression of ET-1 mRNA expression (*p* = 0.5206 while compared to NC) and increased the expression of ET-1 protein with no statistical significance (*p* = 0.0902 while compared to NC). The increased ET-1 mRNA level (**c**) and protein concentration (**d**) mediated by TGF-β1 was enhanced by transfection of PPAR-γ siRNA plasmid with statistical significance (*p < 0.05 while PPARsiRNA + TGF group compared to TGF group). Western blotting showed that PPAR-γ silencing increased the expression of phospho-p38 and phospho-Smad2 (**e**). The results are presented as means ± SD of 3 independent experiments. (NC, negative control group; PPARsiRNA, PPAR-γ silencing group; TGF, TGF-β1 group; PPARsiRNA + TGF, PPAR-γ silencing + TGF-β1 group)

A549 cells were allowed to grow and attain about 80 % confluency and the transfection with PPAR-γ siRNA plasmid or control vectors (negative control) was performed using LipofectamineTM 2000 reagent. Cells were incubated at 37 °C for 24 h in an incubator containing 95 % air: 5 % CO_2_ and full humidity and then treated with 10 ng/mL TGF-β1. The samples were collected and analyzed by real-time qPCR, ELISA and western blotting as described in the materials and methods. The results of RT qPCR and ELISA showed that PPAR-γ silencing reduced the expression of PPAR-γ mRNA, slightly increased the expression of protein with no statistical significance observed, but significantly increased the expression of ET-1 mRNA expression and protein mediated by TGF-β1. PPAR-γ silencing increased the expression of phospho-P38 and phospho-Smad2 according to western blotting analysis (Fig. 5).

### S2505 can increase the expression of PPAR-γ mRNA and suppress the expression of ET-1 mRNA and protein mediated by TGF-β1, decrease the expression of P-P38 and P-Smad2

**Fig. 6:**
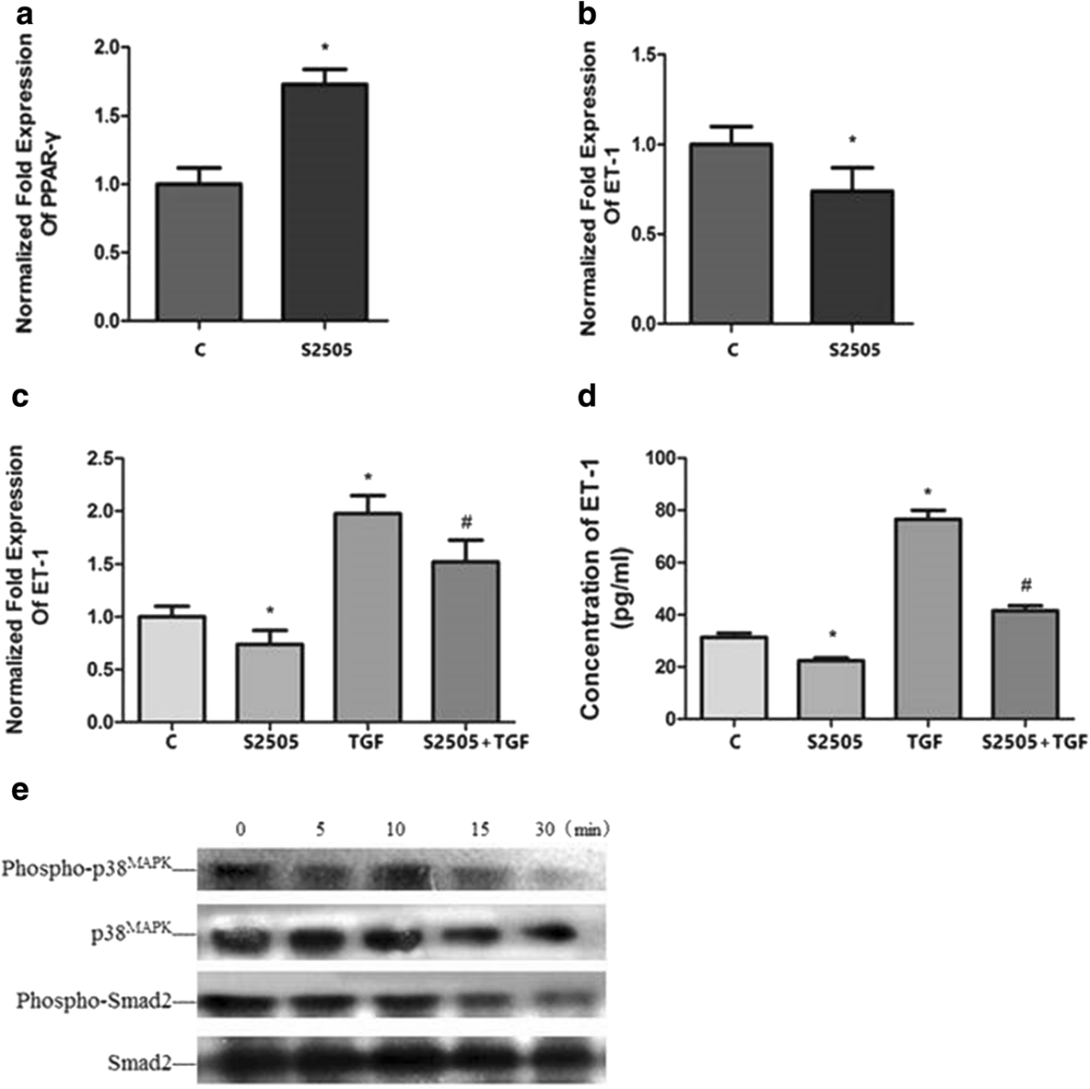
Effect of S2505 on the expression of PPAR-γ and ET-1 mRNA. A549 cells were incubated with 10 ng/mL TGF-β1 or 10 μM S2505 respectively while cells were pre-treated with 10 μM S2505 for 60 min before 10 ng/mL TGF-β1 stimulation for the S2505 + TGF-β1 group. Then the samples were collected and analyzed by real-time qPCR, ELISA and western blotting. The relative PPAR-γ mRNA level (normalized to GAPDH mRNA) in S2505 group was significantly increased (**a**) while compared to control group and the ET-1 level was significantly decreased (**b**) (*p < 0.05). The increased expression of ET-1 mRNA (**c**) and protein (**d**) mediated by TGF-β1was suppressed by S2505 (#*p* < 0.05). Western blotting showed that S2505 decreased the expression of phospho-P38 and phospho-Smad2 (**e**). The results are presented as means ± SD of 3 independent experiments performed in triplicate. (C, control group; S2505, S2505 group; TGF, TGF-β1 group; S2505 + TGF, S2505 + TGF-β1 group)

A549 cells were incubated with PPAR-γ agonist (10 μM S2505) in the presence or absence of 10 ng/mL TGF-β1 and the samples were analyzed by real-time qPCR, ELISA and western blots. The results demonstrated that the PPAR-γ agonist, S2505, increased the expression of PPAR-γ mRNA, reduced the expression of ET-1 mRNA and suppressed the increased expression of ET-1 mRNA and protein mediated by TGF-β1. It also inhibited the expression of phospho-P38 and phospho-Smad2 (Fig. 6).

### PPAR-γ gene over-expression suppressed the expression of ET-1 mRNA and protein mediated by TGF-β1, increase the expression of P-P38 and P-Smad2

**Fig. 7:**
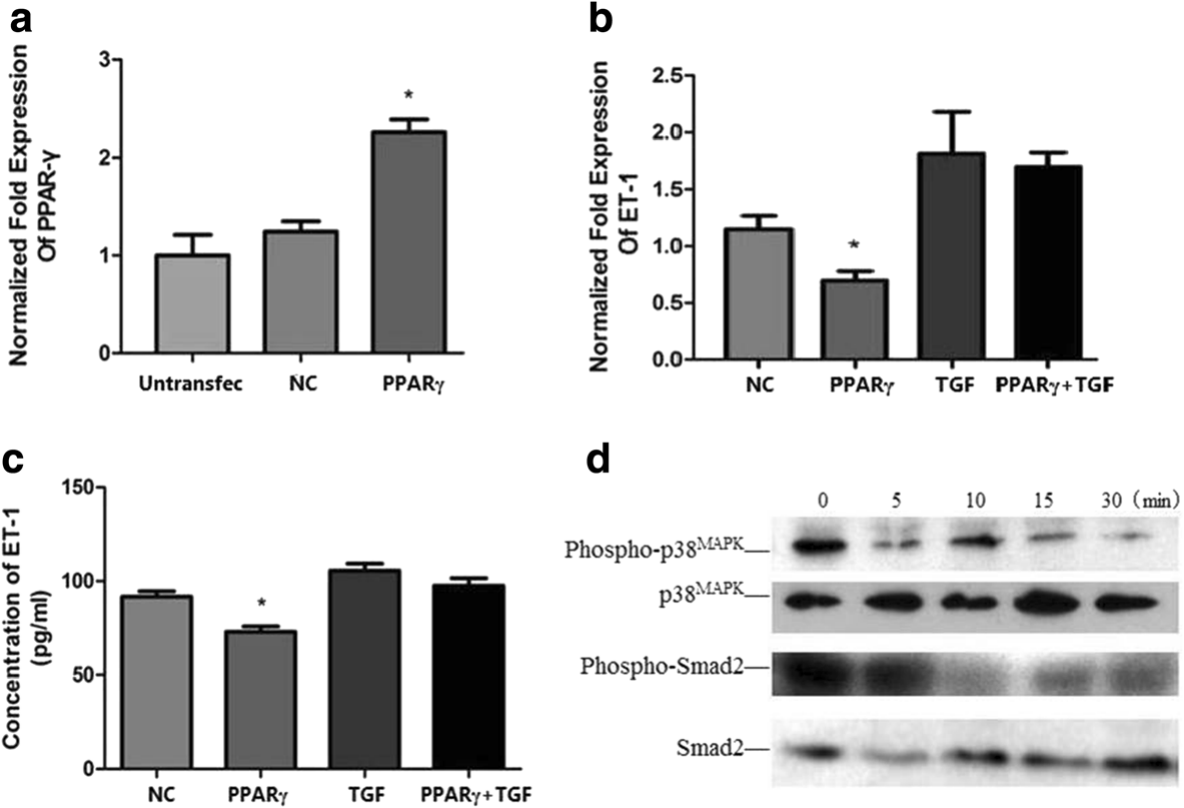
PPAR-γ gene over-expression suppresses the expression of ET-1 mRNA and protein. A549 cells were transfected with PPAR-γ plasmid or control vectors (*negative control*) using LipofectamineTM 2000 reagent and incubated at 37 °C for 24 h in an incubator containing 95 % air, 5 % CO2 and full humidity. The cells were then treated with 10 ng/mL TGF-β1 and the samples were analyzed by real-time qPCR, ELISA and western blotting. The results of real-time qPCR showed that the relative PPAR-γ mRNA level (normalized to GAPDH mRNA) in PPAR-γ transfection group was significantly increased than those in control group (**a**) and ET-1 mRNA level was significantly decreased (**b**) (*p < 0.05). The increased ET-1 mRNA level (**b**) and protein concentration (**c**) mediated by TGF-β1 was suppressed by transfection of PPAR-γ plasmid. Western blotting showed that PPAR-γ gene over-expression decreased the expression of phospho-P38 and phospho-Smad2 (**d**). The results are presented as means ± SD of 3 independent experiments. (NC, negative control group; PPARγ, PPAR-γ plasmid transfection group; TGF, TGF-β1 group; PPARγ + TGF, PPAR-γ plasmid transfection + TGF-β1 group)

A549 cells were allowed to grow till about 80 % confluency and the transfection with PPAR-γ plasmid or control vectors (negative control) was performed using LipofectamineTM 2000 reagent. Cells were incubated at 37 °C for 24 h in an incubator containing 95 % air: 5 % CO_2_ and full humidity and then treated with 10 ng/mL TGF-β1. The samples were collected and analyzed by real-time qPCR, ELISA and western blotting. The results of RT qPCR and ELISA showed that PPAR-γ gene over-expression increased the expression of PPAR-γ mRNA, reduced the expression of ET-1 mRNA as well as the increased expression of ET-1 protein mediated by TGF-β1. PPAR-γ gene over-expression inhibited the expression of phospho-P38 and phospho-Smad2 according to western blots (Fig. 7).

## Discussion

The pathogenesis of PAH still remains elusive, partly because many diseases can lead to PAH and multiple signaling pathways are implicated in this process. There is increasing evidence that the increased expression of ET-1 and the activation of MAPKs are linked to PAH [10, 31]. Interaction of MAPK with TGF-β1 activated signaling cascades were associated with PAH [15, 32]. The serum levels of TGF-β1 were significantly higher in patients with schistosomiasis-associated PAH compared with patients with schistosomiasis but without PAH [33] and TGF-β induced ET-1 expression [34–36]. PPAR-γ is a ligand-activated nuclear receptor and early research suggested that PPAR-γ activators suppressed the ET-1 production both in vitro and in vivo [27, 29, 37]. The way in which TGF-β1 and PPAR-γ regulate the expression of ET-1 and what signaling pathways participate in this process remains unclear. The aim of our study was to investigate the interactions between PPAR-γ, TGF-β1 and ET-1 and the underlying biochemical mechanisms whereby this occurs in A549 cells.

We demonstrated that TGF-β1 could significantly promote the expression of ET-1 mRNA and protein in A549 cells, increase the phosphorylation status of P38 MAPK and Smad2. SB203580 pre-treated A549 cells, resulted in the inhibition of p38 MAPK transduction and nuclear transfer of Smad2 respectively and a suppressive effect on the TGF-β1-induced production of ET-1. These results suggest that both p38 MAPK and Smad2 are involved in TGF-β1 mediated release of ET-1 by A549 cells. These results are consistent with previous studies suggesting that TGF-β1 strongly stimulated the synthesis and secretion of ET-1 in endothelial cells [36, 38], pancreatic stellate cells [35] and lung fibroblasts [34]. TGF-β signaling should be a complex phenomenon and several studies with cell and animal models have reported that TGF-β induced ET-1 expression is mediated through the ALK5/Smad3 and p38 MAPK pathways [36, 39]. TGF-β increased Smad2 and Smad3 phosphorylation and promoted Smad hetero-complex formation and nuclear accumulation in myofibroblasts [40]. Our results demonstrate that TGF-β1, 100 ng/mL BMP-2 and 100 ng/mL BMP-7 increased the relative ET-1 mRNA levels respectively in A549 cells but only the TGF-β1 treatment significantly increased the levels of ET-1 protein expression. There may be several reasons as to why BMP-2 and BMP-7 could not significantly increase the expression of ET-1 protein as well as ET-1 mRNA. The process from ET-1 mRNA to protein expression included translation, post-translational processing and secretion to certain tissues, and multiple factors participate in this process. Any alterations in the various steps in this process would lead to the inconsistencies between mRNA and protein levels while the result of protein expression is probably more reliable index. We investigated the possible pathways by western blot analysis and the results showed that TGF-β1 allowed increases in protein phosphorylation of NF-kB p65, JNK/SAPK but the inhibitors of these pathways could not suppress the ET-1 protein expression when the results of ELISA were considered. It was also found that TGF-β1 allowed increases in protein phosphorylation of p38 MAPK and Smad2 and that SB203580 can effectively inhibit the expression of phspho-p38 MAPK and the nuclear transfer Smad2. Therefore our results strongly suggest that both p38 MAPK and Smad2 were involved in the release of ET-1 mediated by TGF-β1.

Moreover, we have demonstrated that transfecting cells with PPAR-γ siRNA plasmid or pre-treating cells with S2871 increased the expression of phospho-p38 MAPK and phospho-Smad2 and enhanced the expression of ET-1 mRNA and protein mediated by TGF-β1. However, transfecting cells with PPAR-γ plasmid or pre-treating cells with S2505 decreased the expression of phospho-P38 MAPK and phospho-Smad2 and suppressed the expression of ET-1 mRNA and protein mediated by TGF-β1. These results suggest the TGF-induced ET-1 release was dependent on PPAR-γ. Others have shown that PPAR-γ agonist rosiglitazone attenuated ET-1-induced pulmonary vasoconstriction in Sprague-Dawley rats [37, 41–43] and inhibited the activation of p38 MAPK in murine macrophages [44]. Rosiglitazone increased expression of PPAR-γ mRNA and also suppressed the expression of ET-1 mRNA in animal models [28, 45]. When human pulmonary artery endothelial cells were transfected with PPAR-γ siRNA and this was shown to reduce PPAR-γ protein and increased ET-1 protein [46].

In this study, we showed that TGF-β1 promoted the synthesis and secretion of ET-1 in A549 cells, increased the expression of phospho-p38 MAPK and phospho-Smad2 and induced an increase in nuclear transfer of Smad2. SB203580 suppressed the activation of MAPK P38 signal pathway, nuclear transfer of Smad2 and the expression of ET-1. Inhibition of PPAR-γ or PPAR-γ gene silencing suppressed the expression of PPAR-γ mRNA and increased the expression of ET-1 mRNA and protein mediated by TGF-β1 and also increased the expression of phospho-P38 MAPK and phospho-Smad2. However, activation of PPAR-γ or PPAR-γ gene over-expression suppressed the expression of ET-1 mRNA and protein mediated by TGF-β1 and increased the expression of phospho-p38 and phospho-Smad2. These results suggest that the release of TGF-induced ET-1 depended on PPAR-γ through the p38 MAPK and Smad2 pathways. PPAR-γ could, therefore, be considered as a potential therapeutic target for PAH. However, in the present study, we investigated only the interaction between PPAR-γ, TGF-β1 and ET-1 in vitro. Therefore, there is a need to further study the relationship between PPAR-γ, TGF-β1 and ET-1 in vivo and to investigate the anti- vasoconstriction effect of PPAR-γ and its potential role in PAH treatment.

## Conclusions

In conclusion, this study shows that TGF-β1-induced production of ET-1, increased phosphorylation of p38 MAPK and Smad2 nuclear transfer could be prevented by using SB20308 and regulated by PPAR-γ, suggested that p38 MAPK and Smad2 signaling transduction is involved in TGF-β1 induced ET-1 release and that this process was PPAR-γ dependent in A549 cells.

## Abbreviations

CLSM, confocal laser scanning microscopy; ECM, extracellular matrix; ELISA, enzyme-linked immunosorbent assay; ET-1, endothelin-1; mAb, monoclonal antibody; PAH, pulmonary arterial hypertension; PASMC, pulmonary arterial smooth muscle cell; PPAR-γ, peroxisome proliferator-activated receptor gamma; PVR, pulmonary vascular resistance; TGF-β1, transforming growth factor beta 1
